# Calcitriol Analogues Decrease Lung Metastasis but Impair Bone Metabolism in Aged Ovariectomized Mice Bearing 4T1 Mammary Gland Tumours

**DOI:** 10.14336/AD.2018.0921

**Published:** 2019-10-01

**Authors:** Artur Anisiewicz, Beata Filip-Psurska, Agata Pawlik, Anna Nasulewicz-Goldeman, Tomasz Piasecki, Konrad Kowalski, Magdalena Maciejewska, Joanna Jarosz, Joanna Banach, Diana Papiernik, Andrzej Mazur, Andrzej Kutner, Jeanette A Maier, Joanna Wietrzyk

**Affiliations:** ^1^Department of Experimental Oncology, Hirszfeld Institute of Immunology and Experimental Therapy, Wroclaw, Poland.; ^2^Faculty of Veterinary Medicine, Wroclaw University of Environmental and Life Sciences, Wroclaw, Poland.; ^3^Masdiag Sp. z o.o., Żeromskiego 33, Warsaw, Poland.; ^4^Université Clermont Auvergne, INRA, UNH, Unité de Nutrition Humaine, F-63000 Clermont-Ferrand, France.; ^5^Pharmaceutical Research Institute, 01-793 Warsaw, Poland.; ^6^Università di Milano, Dept. Biomedical and Clinical Sciences, 20157 Milano, Italy

**Keywords:** breast cancer, elderly, ovariectomized mice, metastasis, calcitriol, vitamin D analogues

## Abstract

Calcitriol and its analogues are considered drugs supporting the anticancer treatment of breast cancer and preventing the osteoporosis that results from the development of cancer or from chemotherapy or hormone therapy. Following the orthotopic implantation of 4T1 mammary carcinoma cells into aged ovariectomized (OVX) mice, we evaluated the effects of calcitriol and its two analogues, PRI-2191 and PRI-2205, on metastatic spread and bone homeostasis. Calcitriol and its analogues temporarily inhibited the formation of metastases in the lungs. Unexpectedly, only mice treated with calcitriol analogues showed a deterioration of bone-related parameters, such as bone column density, marrow column density and the CaPO_4_ coefficient. These findings correlated with an increased number of active osteoclasts differentiated from bone marrow-derived macrophages in mice treated with the analogues. Interestingly, in the tumours from mice treated with PRI-2191 and PRI-2205, the expression of *Tnfsf11* (RANKL) was increased. On the other hand, osteopontin (OPN) levels in plasma and tumour tissue, as well as TRAC5b levels in tumours, were diminished by calcitriol and its analogues. Despite a similar action of both analogues towards bone metabolism, their impact on vitamin D metabolism differed. In particular, PRI-2191 and calcitriol, not PRI-2205 treatment significantly diminished the levels of both 25(OH)D_3_ and 24,25(OH)_2_D_3_. In conclusion, though there is evident antimetastatic activity in old OVX mice, signs of increased bone metabolism and deterioration of bone mineralization during therapy with calcitriol analogues were observed.

There is ample evidence that cancer is linked to the ageing process (e.g., reviewed in [1]). In animal studies, Meehan et al. have shown that ageing influences the metastatic phenotype of cancer cells. Indeed, lung metastases are less aggressive and smaller in old mice than in young mice [2]. Moreover, impairment of angiogenesis is described to be the cause of slower cancer growth in the elderly [3]. Further, tumour responsiveness to therapeutic agents differs in young and old animals [3,4]. Despite this evidence, the overwhelming majority of research focusing on the antitumour activity of various compounds is carried out on young animals.

Vitamin D deficit is ubiquitous and is more common and severe in the elderly [5]. In addition to the classical effects associated with vitamin D deficiency, the non-classical ones are common, such as an increased risk of sepsis, cardiovascular and metabolic disorders (e.g., hyperlipidaemia, type 2 diabetes, dementia, stroke and heart failure) [6]. The hypovitaminosis D often correlates with an increased risk of breast cancer, its prognosis, and sensitivity to antineoplastic therapy [7,8]. Furthermore, cancer itself [9] and the applied therapeutic schemes that reduce oestrogens level (e.g., oophorectomy, chemo-therapy, and aromatase inhibitors) enhance bone resorption without a compensatory increase in bone formation [10-13].

1,25-Dihydroxyvitamin D_3_ (calcitriol), a hormonally active metabolite of vitamin D_3_, is the ligand for the nuclear vitamin D_3_ receptor (VDR). VDR is expressed and regulated in the mammary gland during the reproductive cycle [14]. Moreover, in VDR-knockout mice compared to wild-type mice, enhanced mammary gland development during puberty and early pregnancy, as well as weaker apoptosis during involution, were observed [14]. Further, chronic removal of VDR signalling during ageing revealed large effects on ovarian function and metabolism that are associated with mammary gland alterations, such as atrophy of the fat pad, epithelial ductal branching degeneration, extended lymph nodes and chronic inflammation [15]. Recently, Chen et al. reported that older mice (1 year old) develop different types of spontaneous tumours during vitamin D deficiency [16]. Moreover, the same authors have shown that vitamin D deficiency leads to increased oxidative stress and DNA damage, resulting in accelerated ageing and tumourigenesis through oncogene activation [16].

Calcitriol and its analogues have been shown to influence the proliferation of stromal and epithelial mammary gland cells* in vitro*. Moreover, their inhibitory effect on the growth of different breast cancer cell lines has also been demonstrated [17-19]. Additionally, there are experimental animal studies suggesting the beneficial influence of vitamin D or its analogues on the growth of breast cancer xenografts [17,20,21] and transplantable mouse mammary gland tumours [22-24]. The animal studies also showed that vitamin D deficiency accelerated mammary gland tumour growth and metastasis [25,26]. On the other hand, our recent studies have shown that in the 4T1 mouse mammary gland cancer model transplanted orthotopically, the metastatic spread of cancer cells was accelerated upon treatment with calcitriol or its analogues in young 6- to 8-week-old mice [27,28]. Cao et al. also demonstrated enhanced 4T1 primary tumour growth in mice treated with vitamin D_3_ [29]. There are also randomized clinical trials showing that vitamin D supplementation does not significant effect against cancer [30]. Moreover, the clinical trials in which low calcaemic analogues of calcitriol are used in cancer patients as monotherapy do not prove its efficacy [31,32]. More benefits seem to result from the combined use of calcitriol or its analogues with other anticancer therapies (summarized in recent review [31]). For example, inecalcitol combined with docetaxel in prostate cancer [33], or with decitabine in acute lymphatic leukemia (AML) has been subjected to such clinical trials. Ultimately, inecalcitol was approved as an orphan drug in the treatment of AML [31]. The lack of benefits from vitamin D and its analogues in clinical trials is in contrast with the encouraging effects from preclinical studies and could be due to undefined bias in patient selection criteria or to the fact that preclinical studies were performed in inadequate experimental models (young animals only).

The majority of breast cancer patients are over 40 years old, and the survival outcomes of older and younger breast cancer patients are different [34]. Moreover, it has been postulated to supplement with vitamin D to avoid deficiency in breast cancer patients and in the elderly [35-38]. The study presented here focuses on the assessment of the effect of vitamin D and its selected analogues on the growth and metastasis of 4T1 tumours in 60-week-old ovariectomized (OVX) mice.

## MATERIALS AND METHODS

### Compounds

Calcitriol (1,25(OH)_2_D_3_) and its analogues PRI-2191 and PRI-2205 were obtained from the Pharmaceutical Research Institute, Warsaw, Poland. Samples of the compounds were stored in amber ampoules under argon at -20°C. Compounds were dissolved in 99.8% ethanol and then diluted in 80% propylene glycol (PEG) to reach the required concentrations. All compounds were freshly prepared immediately before administration. Working solutions of macrophage colony-stimulating factor (M-CSF; BioLegend, San Diego, USA) and receptor activator for nuclear factor κB (RANKL, R&D Systems, Inc., Minneapolis, USA) used in osteoclast formation were prepared and stored according to the manufacturer’s instructions.

### Cells and cell line culture

Mouse mammary adenocarcinoma 4T1 cells were obtained from American Type Culture Collection (ATCC, Rockville, Maryland, USA). The cells were immediately used in experiments. The 4T1 cell line was maintained in a mixture of RPMI-1640 and Opti-MEM (1:1, Gibco, Scotland, UK) adjusted to contain 3.5 g/l glucose, 2 mM L-glutamine and 0.5 mM sodium pyruvate (Sigma-Aldrich Chemie GmbH, Steinheim, Germany) with 5% foetal bovine serum (HyClone, GE Healthcare, UK). Culture medium was supplemented with 100 U/mL penicillin (Polfa Tarchomin S.A., Warsaw, Poland) and 100 µg/ml streptomycin (Sigma-Aldrich Chemie GmbH, Steinheim, Germany). The cells were grown at 37°C in a humidified atmosphere with 5% CO_2_.

### Mouse studies

Female BALB/c mice at 60 weeks old and weighing 24-34 g were obtained from the Center of Experimental Medicine of the Medical University of Bialystok (Bialystok, Poland) and were maintained in specific pathogen-free conditions. All experiments were performed according to *EU Directive 2010/63/EU* on the protection of animals used for scientific purposes and were approved by the first Local Committee for Experiments with the Use of Laboratory Animals, Wroclaw, Poland (Permission No.: 40/2014 from July 16, 2014).

Ovariectomy (OVX) was performed under general anaesthesia. Mice received an intraperitoneal injection of buprenorphine at a dose of 0.2 mg/kg (VET-AGRO Sp. z o.o., Poland) and were anaesthetized with a mixture of synthetic air and isoflurane (4% for induction and 2-3% v/v for maintenance; 200 ml/min; Aerrane isofluranum, Baxter, Canada). After shaving, a 1-2 cm skin middle incision was made, the skin was gently separated, and two small incisions were made lateral to the midline. The ovaries were identified and cut, and the wounds were closed with soluble surgical sutures. Twenty-four hours after surgery and on subsequent days when signs of pain existed, buprenorphine (0.1 mg/kg) was injected. As controls, mice underwent the same surgical procedure without removing the ovaries (sham).

Four weeks after OVX and sham operations, BALB/c mice received an orthotopic inoculation of 1×10^4^ viable 4T1 breast cancer cells per mouse in 0.05 ml (45 μl of Hanks solution with 5 μl of Matrigel) into the right mammary fat pad and then were randomly divided into groups to receive treatment (7 mice per group). The day of tumour inoculation was labelled as day 0.

**Figure 1.**
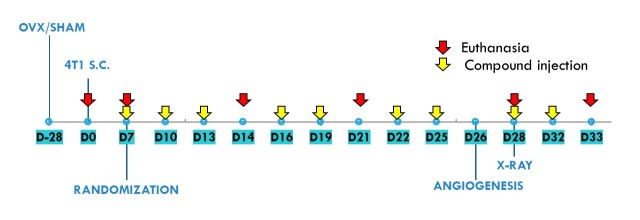
Time course of experiment.

Vitamin D analogues were administered subcutaneously (s.c.) thrice weekly starting from day 7 after tumour cell inoculation. The single dose of compounds was as follows: calcitriol, 0.5 µg/kg; PRI-2191, 1.0 µg/kg; and PRI-2205, 10.0 µg/kg (Fig. 1). The doses of compounds were selected during previous studies based on their toxicity and anticancer activity [39]. Control mice (both OVX and sham operated) were administered with vehicle (80% PEG) in the same volume and scheme as vitamin D compounds. Mice were euthanized as follows: (1) Sham operated: day 0 (before tumour cell transplantation); day 7 (before compound administration); and days 21 and 33; (2) OVX: day 0 (before tumour cell transplantation); day 7 (before compound administration); and days 14, 21, 28 and 33.

In the graphs, control mice (receiving the vehicle) are described as follows: "sham" - sham operated, tumour bearing mice; "control" - OVX, tumour bearing mice. Day 0 means mice not inoculated with cancer cells, sham-operated or OVX.

Blood and tumours were harvested on days 14, 21, 28, and 33 after the inoculation of 4T1 cells, and bone marrow cells were collected on day 28. Blood was also collected before tumour cell inoculation (day 0) and on day 7, before starting treatment. The mice were anaesthetized with a 3-5% (v/v) mixture of isoflurane (Aerrane isofluranum, Baxter, Canada) in synthetic air (200 ml/min) prior to blood collection and sacrifice. The control group euthanized on day 7 was included to monitor the condition of the tumour-bearing mice at the beginning of the treatment. Some of the analyses were performed only on day 14 or 21 (early stages of tumour progression) and on day 28 or 33 (late stages of tumour progression) (Fig. 1).

Radiographs of mouse bones were taken from old (60 week) and young (6-8-week-old) BALB/c mice orthotopically inoculated with 4T1 breast cancer cells (1×10^4^ cells per mice) during the studies as described previously [27,28]. The protocol for young mice was also approved by the first Local Committee for Experiments with the Use of Laboratory Animals, Wroclaw, Poland (Permission no.: 40/2014 from July 16, 2014).

Tumours were measured by using a calliper three times a week. Tumour volume was calculated using the formula (*a*^2^ × *b*)/2, where *a* = shorter tumour diameter in mm and *b* = longer tumour diameter in mm. Mice were sacrificed when the tumour volume exceeded 2,000 mm^3^. Multiple tumours were not observed in our study. Metastases were counted in lungs fixed in 4% buffered formalin by two independent examiners.

### Evaluation of tumour angiogenesis

To evaluate the influence of calcitriol and its analogues on tumour angiogenesis, tumour perfusion was evaluated on day 24 by ultrasound imaging, as previously described [27]. Briefly, ultrasound imaging was performed after MicroMarker^TM^ Contrast Agent (VisualSonics, Canada) intravenous injection in anaesthetized animals placed on the handling station. The central section of the tumour was visualized. Signal of the contrast marker accumulating in the tumour was recorded using the probe at 13-24 MHz frequency (MS250, VisualSonics, Canada). Next, the peak-enhancement [Contrast Mean Power as l.a.u.] parameter was calculated using Vevo LAB 1.7.1 Software (VisualSonics, Ontario, Canada).

### Bone homeostasis

Calcium level was measured in each sample of plasma using the Cobas c 111 z ISE device (Roche Diagnostics Ltd., Rotkreuz, Switzerland).

Radiographs of mouse bones were taken on day 28 after the administration of anaesthesia on a Kodak/ Carestream FX PRO Imaging System for a total of 3-6 young and old mice (for each group). Images were acquired using the X-ray source energy at 35 kVp with an X-ray calibrated energy of ~13 keV. Binning was set at 2 X & 2 Y with a 0.4 mm aluminium filter, an ƒ-stop of 5.57, an exposure time of 2 min, and FOV = 200, which provided sufficient grey scale values for acquiring an image quality suitable for bone mineral density measurements. Images were processed using Bruker Molecular Imaging software v.7.1.3.20550 with the Bruker Bone Density Software Module by quantifying pixels after selecting the femur regions of interest [40,41].

The analysis of the vitamin D metabolite profile (25(OH)D_3_, 25(OH)D_2_ and 24,25(OH)_2_D_3_) was performed with high-performance liquid chromatography coupled with mass spectrometry using an isotope dilution methodology. Whole blood was collected on Whatman 903 cards and dried to obtain dried blood spots (DBS). Two 3 mm discs were cut out, pooled together and subjected for methanol extraction. Before analysis, vitamin D metabolites were derivatized with 4-(4'-dimethyl-aminophenyl)-1,2,4-triazoline-3,5-dione (DAP TAD) [42]. Chromatographic separation (Exion LC, Sciex) was performed on a Kinetex 1.7 µm F5 100 Å, 50 x 2.1 mm column in 8-min gradients of water and acetonitrile with 0.1% formic acid (0.45 mL/min; 40°C). Detection in MRM mode was done on a 4500QTRAP (Exion LC, Sciex) in electrospray positive ionization mode. The results were multiplied by haematocrit values to obtain vitamin D metabolite serum concentrations.

### Mouse T Helper Cell Differentiation RT^2^ Profiler Array

Total RNA was isolated from tumour tissue using TRIzol (TRI Reagent; Sigma-Aldrich, Germany) according to the manufacturer’s procedure. The quantity of RNA was established using a NanoDrop 2000 at 260 nm (Thermo Fisher Scientific, Waltham, MA, USA). First-strand cDNA synthesis was performed using a compatible RT First Strand Kit (Qiagen, Hilden, Germany), eventually achieving 0.5 µg of cDNA (four mice pooled per group) for a single reaction. The Mouse T Helper Cell Differentiation RT^2^ Profiler Array was obtained from Qiagen Company (Qiagen, Hilden, Germany). This PCR array detects the expression of 84 key genes associated with differentiation of T cell precursors into specific effector subtypes and five housekeeping genes (list of genes included in Supplementary Table 1). Cycling conditions for PCR amplification were as follows: 95°C for 10 min (1 cycle), followed by 40 cycles of 95°C for 15 s and 60°C for 1 min. The expression of mRNA was measured by real-time PCR through a ViiA™ 7 Real-Time PCR System (Thermo Fisher Scientific, USA) with SYBR green chemistry (Qiagen, Hilden, Germany). Relative quantification (RQ) values of target genes was defined using ΔΔCt values with reference to the heat shock protein 90 alpha, class B member 1 (*Hsp90ab1*) gene. Data were analysed using Qiagen online software suitable for the purchased kit (Qiagen, Hilden, Germany).

### ELISA

The expression of selected proteins was detected in mouse plasma and tumour homogenates. Blood specimens were collected during mice euthanasia. Next, blood was immediately centrifuged at 2000 x g for 15 min at 4°C, and plasma was transferred into fresh tubes. Tumour lysates were prepared as described previously [27]. For the quantitative determination of 17-β-estradiol, osteopontin (OPN), receptor activator for nuclear factor κB ligand (RANKL), osteocalcin and tartrate-resistant acid phosphatase 5b (TRACP5b), ELISA kits were used according to the manufacturers’ instructions (Demeditec Diagnostics GmbH, Germany; R&D Systems, Inc., Minneapolis, Canada; Elabscience, Houston, USA; and Cusabio, Houston, USA; respectively).

**Figure 2.**
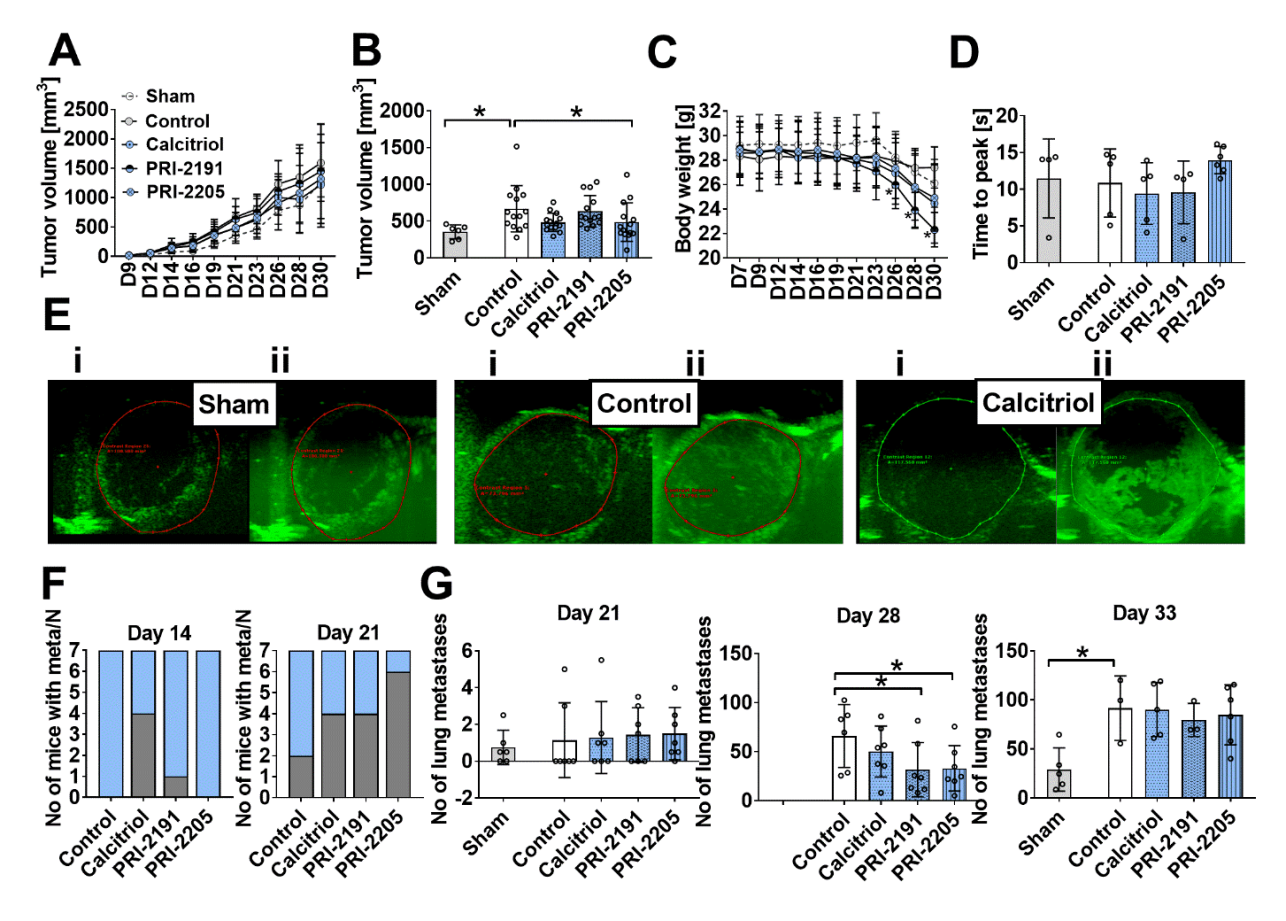
The effect of calcitriol and its analogues on tumour progression in aged OVX mice. (A) Kinetics of tumour growth. (B) Tumour volume measured on the 21^st^ day of the experiment. (C) Body weight kinetics. (D) Tumour blood perfusion estimated by ultrasound imaging as time to peak values measured on day 24 after 4T1 mammary gland inoculation. (E) Representative images of tumour perfusion in sham operated mice and OVX mice treated with vehicle or OVX mice treated with calcitriol (i) before and (ii) after contrast agent injection. (F) The number of mice diagnosed with metastases; graphs represent the number of mice with macroscopic metastatic nodules on days 14 and 21 (grey) and the number of mice in the group (N, blue). On the next days of observation in all animals, metastatic nodules were detected. (G) The number of lung metastases counted in fixed lungs on days 21, 28 and 33. Mice were ovariectomized (OVX) or sham operated (Sham); after 4 weeks, they were inoculated orthotopically with 4T1 cells (day 0). From day 7 (7 days after tumour inoculation), vitamin D analogues or vehicle were administered subcutaneously (s.c.) thrice weekly. The single doses of compounds were as follows: calcitriol, 0.5 µg/kg; PRI-2191, 1.0 µg/kg; and PRI-2205, 10.0 µg/kg. The number of mice analysed was 4-7 per group. Control mice (receiving the vehicle) are described as follows: "sham" - sham operated, tumour bearing mice; "control" - OVX, tumour bearing mice. Data presentation: (A) and (C) mean with standard deviation (SD); (B), (D) and (G) data for individual animals with mean and (SD). Statistical analysis: Kruskal-Wallis multiple comparison test. *P<0.05.

**Figure 3.**
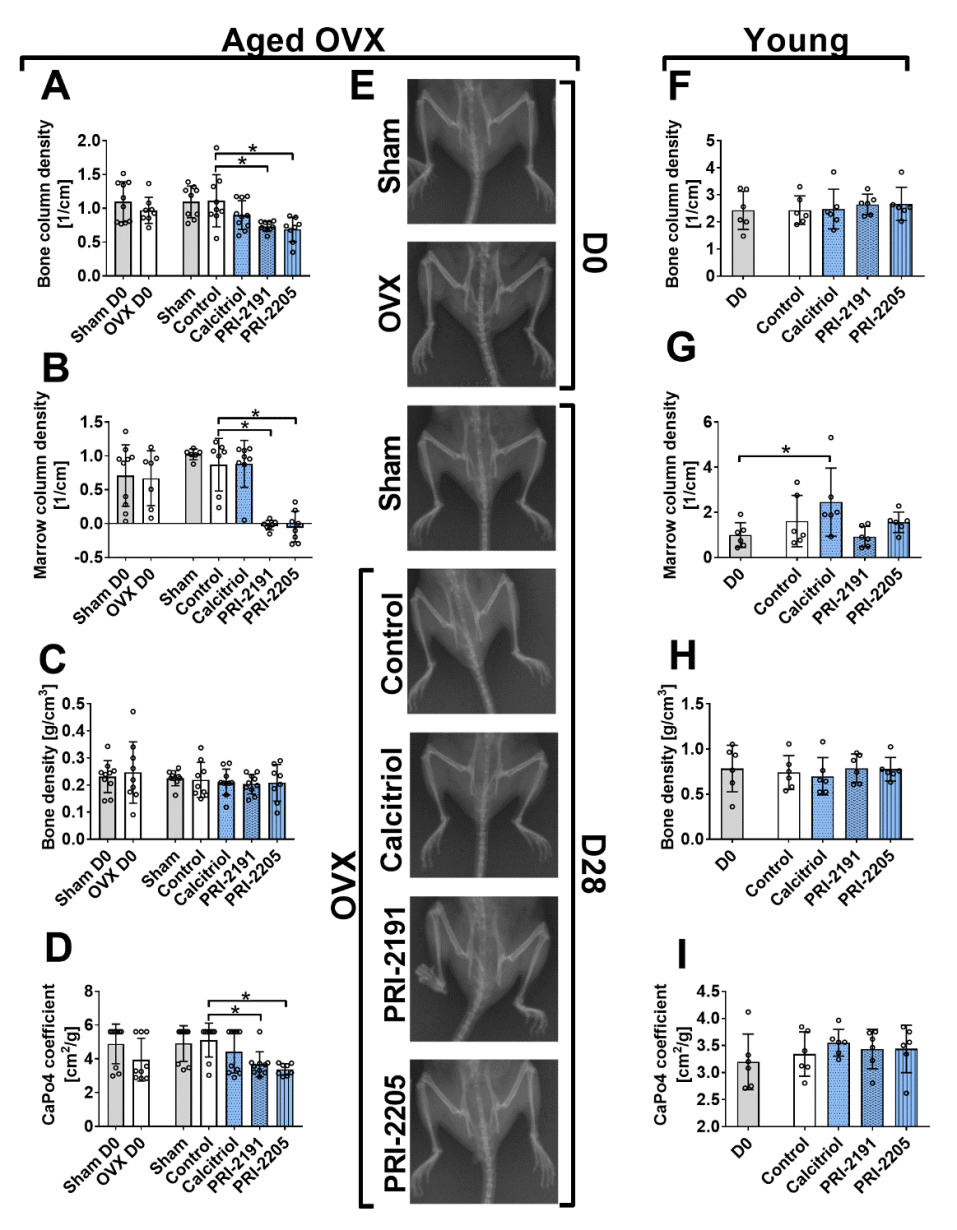
The influence of calcitriol and its analogues on bone mineralization in mice bearing 4T1 tumours (day 0 and day 28). (A-E) Analysis performed on aged OVX mice. (F-I) Analysis performed on young mice. (A) and (F) Bone column density (corrected X-ray extinction for the cylindrical bone). (B) and (G) Marrow column density (corrected X-ray extinction for the marrow). (C) and (H) Bone density. (D) and (I) CaPO_4_ coefficient. (E) Representative X-ray pictures of aged OVX mice. Bruker In-Vivo FX PRO imaging equipped with an X-ray unit was used. Mice were ovariectomized (OVX) or sham operated (Sham), and after 4 weeks, they were inoculated orthotopically with 4T1 cells (day 0). From day 7 (7 days after tumour inoculation), vitamin D analogues or vehicle were administered subcutaneously (s.c.) thrice weekly. The single doses of compounds were as follows: calcitriol, 0.5 µg/kg; PRI-2191, 1.0 µg/kg; and PRI-2205, 10.0 µg/kg. Both femurs of 3-6 mice were used for analysis. Control mice (receiving the vehicle) are described as follows: "sham" - sham operated, tumour bearing mice; "control" - OVX, tumour bearing mice. Day 0 means mice not inoculated with cancer cells, sham-operated or OVX. Statistical analysis: Kruskal-Wallis multiple comparison test. *p<0.05 compared to control mice on the relevant day of treatment or as indicated.

### Ex vivo assay for osteoclastogenesis

To prepare bone marrow-derived macrophages (BMDMs), bone marrow cells were collected from the tibiae and femurs of mice on day 28 of the experiment. After this, the cells were frozen in 90% foetal bovine serum (HyClone, GE Healthcare, UK) with 10% DMSO (Sigma-Aldrich Chemie GmbH, Steinheim, Germany). Then, 10×10^6^ thawed cells were seeded on the non-tissue culture-treated Petri dishes (Sarstedt, Numbrecht, Germany) in 10 ml of MEM-α (Thermo Scientific, Waltham, USA) containing 20 ng/ml M-CSF. Every other day, media were replaced with fresh media containing 20 ng/ml M-CSF, and cultures were maintained for 7 days. Next, differentiated BMDMs were detached from dishes by trypsin, and 2×10^4^ BMDMs were seeded in 24-well plates (Corning, New York, USA) in 500 µl of MEM-α supplemented with 20 ng/ml M-CSF and 20 ng/ml RANKL. Media were changed every other day, and cultures were maintained for 7 days. After this, the stain for active osteoclasts was prepared using an Acid Phosphatase Leukocyte (TRAP) Kit according to manufacturer’s instructions. Microscopic examination and photographs were captured using a bright field microscope (Olympus IX81) connected to a camera equipped with Olympus Stream Image Analysis software (Olympus Europe Holding GmBH, Hamburg, Germany). Blinded microscopic examination was performed at 10× magnification, and photographs were taken at 4× magnification. Characterized by stained acid phosphatase (dark purple colour visible in the cytoplasm of cells) and numerous cell nuclei (>5), active osteoclasts were counted over the entire surface of the well. The percentage of active osteoclasts in the study groups was compared to the values obtained for the control group.

### Statistical evaluation

Statistical analysis was performed using GraphPad Prism 7.01 (GraphPad Software Inc., USA). Shapiro-Wilk’s normality test and Bartlett’s test were used to check the assumptions for analysis of variance (ANOVA). Tests used for each data analysis are indicated in the figure legends. *P* < 0.05 was considered significant.

## RESULTS

### Calcitriol and its analogues transiently decreased the metastatic spread of 4T1 breast carcinoma without influencing primary tumour growth or angiogenesis in old OVX mice

Initially, we evaluated whether calcitriol and selected analogues affected the growth of primary tumours and the degree of tumour vascularization in aged OVX mice. We measured the volume of the tumours and found that the examined compounds did not influence primary tumour growth at any of the times tested (Fig. 2A), with the exception of PRI-2205, which decreased tumour volume on day 21 (Fig. 2B). Accordingly, angiogenesis investigated by ultrasound imaging on day 24 was unaffected (Fig. 2D and E).

Next, we analysed how many mice had macroscopic lung metastatic foci and found that 4 of 7 calcitriol-treated mice showed metastases after 14 days of treatment, whereas no metastases were detectable in control 4T1 tumour bearing mice (Fig. 2F). On day 21, the number of mice with metastases was markedly higher in the groups treated with calcitriol and analogues than in the control groups (Fig. 2F). It is noteworthy that while the mean number of metastatic foci did not differ between the control and treated groups after day 21 (Fig. 2G), we observed a significant inhibition of lung colonization after 28 days of treatment with PRI-2191 and PRI-2205. The influence on metastasis by calcitriol on day 28 was not statistically significant. At the end of the experiment (day 33), the effect on lung metastasis was no longer observed (Fig. 2G). The tumour volume and number of metastases were lower in sham-operated mice than in control OVX mice (Fig. 2A, B and G).

### Calcitriol and analogues increased the tumour growth-associated decrease in body weight

Starting from day 23, the body weight of all mice decreased as a result of tumour progression. This effect was particularly evident in ovariectomized mice treated with calcitriol, PRI-2191 (p<0.05 starting from day 26) and PRI-2205 (Fig. 2C). Mice were sacrificed when their body weight decreased by more than 20% in the interval between two consecutive measurements or when mice showed clear symptoms of disease, such as changes in behaviour, posture, and appearance or tumours larger than 2000 mm^3^ or with necrotic spots. In the control group, 1 mouse was euthanized on day 26, and 3 were sacrificed on day 29. In the calcitriol-treated group, 2 mice were euthanized on day 30. In the group treated with PRI-2191, 4 mice were euthanized on day 31. In the PRI-2205-treated group, only 1 mouse was sacrificed on day 32.

### Both calcitriol analogues influenced bone mineralization in aged OVX mice

PRI-2191 and PRI-2205, not calcitriol, decreased bone column density (Fig. 3A), marrow column density (Fig. 3B) and the CaPO_4_ coefficient (Fig. 3D) in aged OVX mice, while they had no effect in young mice (Fig. 3F-I).

**Figure 4.**
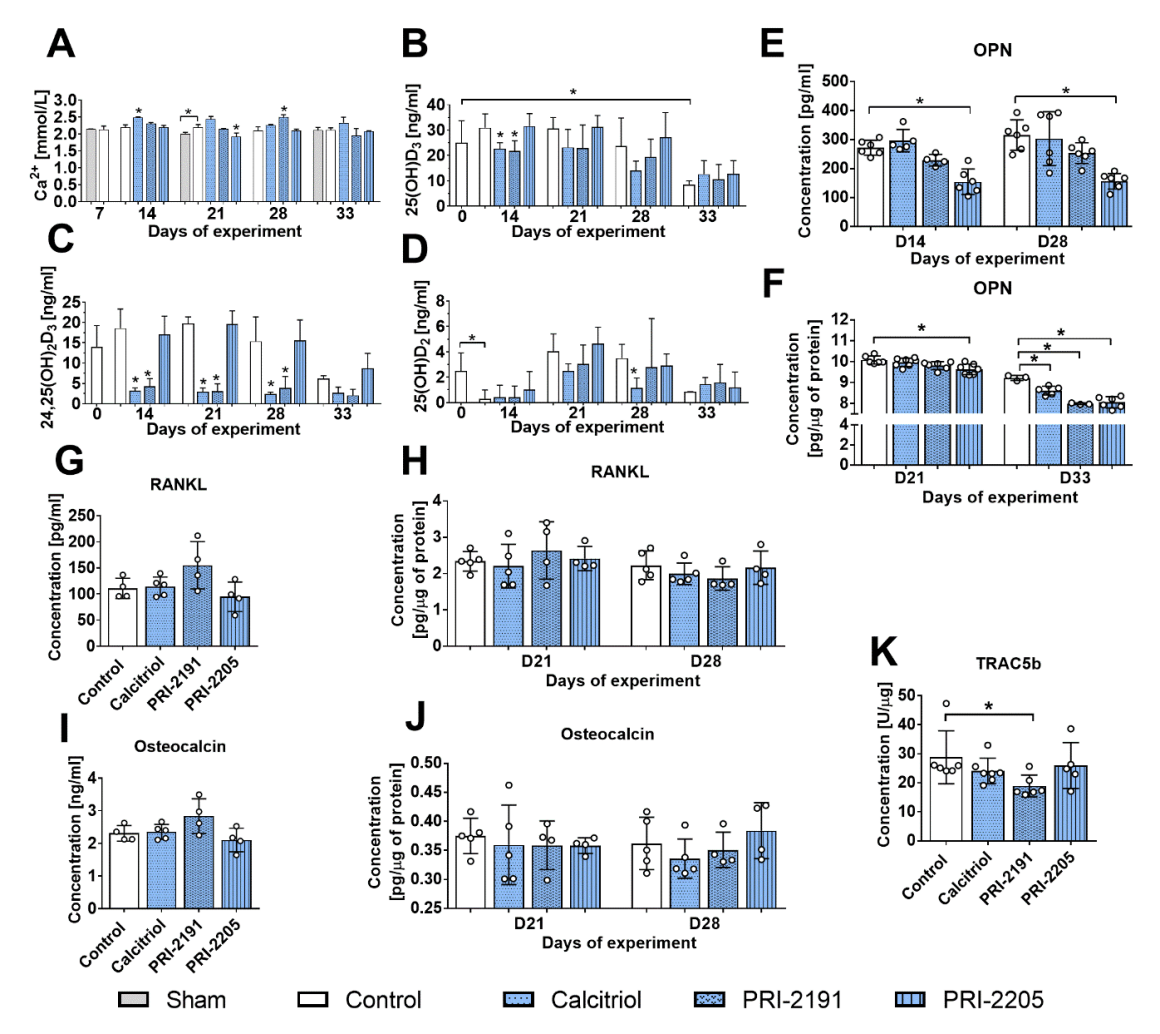
Ca^2+^ plasma concentration and vitamin D metabolites in the blood; plasma and tumour cytokine levels in aged OVX mice bearing 4T1 mammary gland tumours and treated with calcitriol and its analogues. (A) Plasma concentration of Ca^2+^. The amount of vitamin D metabolites in whole blood: (B) 25(OH)D_3_, (C) 24,25(OH)_2_D_3_, (D) 25(OH)D_2_. (E) Osteopontin (OPN) level in mouse plasma and (F) tumour tissue. Plasma concentration of (G) RANKL and (I) osteocalcin. Tumour tissue levels of (H) RANKL, (J) osteocalcin, (K) TRAC5b. Mice were ovariectomized (OVX) or sham operated (Sham); after 4 weeks, they were inoculated orthotopically with 4T1 cells (day 0). From day 7 (7 days after tumour inoculation), vitamin D analogues or vehicle were administered subcutaneously (s.c.) thrice weekly. The single doses of compounds were as follows: calcitriol, 0.5 µg/kg; PRI-2191, 1.0 µg/kg; and PRI-2205, 10.0 µg/kg. Number of samples analysed per group: 3-7. Control mice (receiving the vehicle) are described as follows: "sham" - sham operated, tumour bearing mice; "control" - OVX, tumour bearing mice. Day 0 means mice not inoculated with cancer cells, sham-operated or OVX. Statistical analysis: Kruskal-Wallis multiple comparison test. *P<0.05 compared to control mice on the relevant day of treatment or as indicated.

The calcium plasma concentration increased significantly after treatment with calcitriol (day 14) and PRI-2191 (day 28), whereas PRI-2205 diminished the Ca^2+^ level on day 21 (Fig. 4A). Vitamin D metabolites were also measured in mouse whole blood (Fig. 4B-D). Both calcitriol and PRI-2191 decreased the level of 25(OH)D_3_ on days 14 (p<0.05) and 21 (Fig. 4B). During the course of the experiment, calcitriol and PRI-2191 significantly decreased the blood level of 24,25(OH)_2_D_3_ (Fig. 4C). Calcitriol also decreased the level of 25(OH)D_2_ on days 21 and 28 (Fig. 4D). PRI-2205 did not significantly affect the level of plasma vitamin D metabolites (Fig. 4B-D).

**Figure 5.**
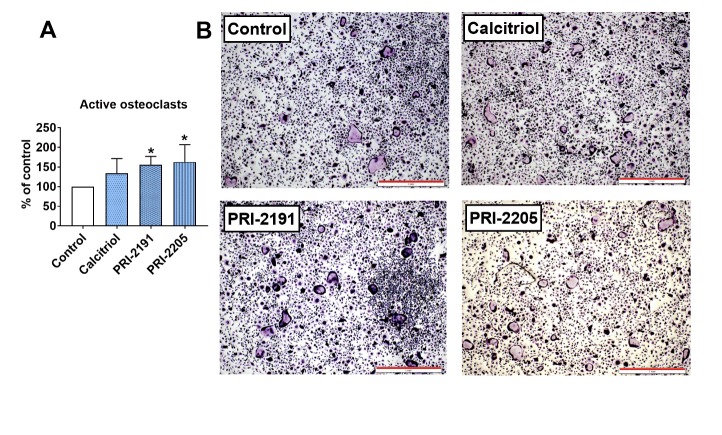
*Ex vivo* osteoclastogenesis of bone marrow macrophages in aged OVX mice bearing 4T1 mammary gland tumours and treated with calcitriol or its analogues. (A) Active osteoclasts. TRAP-positive cells characterized by acid phosphatase staining (dark purple colour visible in the cytoplasm of cells) and more than five nuclei. BMDMs derived from mice on day 28 of the experiment were cultured with M-CSF (20 ng/ml) and RANKL (20 ng/ml). Mice were ovariectomized (OVX) or sham operated (Sham); after 4 weeks, they were inoculated orthotopically with 4T1 cells (day 0). From day 7 (7 days after tumour inoculation), vitamin D analogues or vehicle were administered subcutaneously (s.c.) thrice weekly. The single doses of compounds were as follows: calcitriol, 0.5 µg/kg; PRI-2191, 1.0 µg/kg; and PRI-2205, 10.0 µg/kg. Data are presented as the mean and standard deviation (SD). The percentage of cells compared to control mice is presented. The number of samples analysed was 4 per group. "Control" means OVX, tumour bearing mice. Statistical analysis: Sidak’s multiple comparison test. *P<0.05 compared to control mice. (B) Representative microphotographs of TRAP-positive osteoclasts. Scale bar = 1 mm.

### Calcitriol and its analogues significantly decreased the level of OPN in the tumour tissue of aged OVX mice bearing 4T1 tumours.

PRI-2205 significantly decreased the plasma level of OPN on days 14 and 28, and a similar trend was noted for PRI-2191, although it did not reach statistical significance (Fig. 4E). The analysis of cytokines in tumour tissue showed a marked decrease in OPN on day 33 by all compounds used (Fig. 4F). The concentrations of receptor activator of NF-κB ligand (RANKL) and osteocalcin in mouse plasma were measured on days 21 and 28. On day 21, treatment did not change the level of either protein (data not shown). A slight but not statistically significant increase in RANKL and osteocalcin levels was observed after 28 days of treatment with PRI-2191 (Fig. 4G and I, respectively). On day 21, RANKL showed a tendency (*p*=0.0623) to be increased in tumour tissue from mice treated with PRI-2191 (Fig. 4H). The osteocalcin tumour tissue level did not change significantly (Fig. 4J), but TRAC5b was significantly decreased by PRI-2191 (day 28) (Fig. 4K).

### PRI-2191 and PRI-2205 increased the ex vivo differentiation of bone marrow macrophages to active osteoclasts in aged OVX mice

The number of active osteoclasts generated from bone marrow-derived macrophages (BMDMs) was significantly increased in mice treated with both calcitriol analogues but not with calcitriol (Fig. 5A and B).

### Gene expression profile of tumour tissue harvested from aged OVX mice

**Figure 6.**
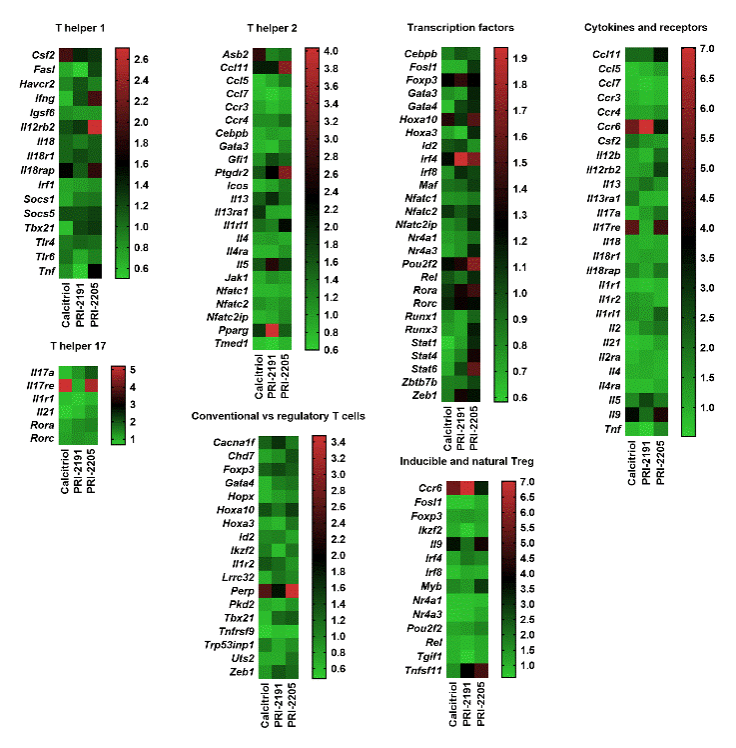
Heat maps of tumour tissue real-time PCR screening performed using the *Mouse T Helper Cell Differentiation RT^2^ Profiler Array*. Real-time PCR screening was performed using tumour tissue harvested on day 21. Data are presented as the mean fold change. The fold change (RQ) of target genes was defined using the double delta Ct method in reference to the heat shock protein 90 alpha (cytosolic), class B member 1 (*Hsp90ab1*). The results were adjusted to the values obtained for the control group (OVX, tumour-bearing, vehicle treated). Data analysis was performed using Qiagen online software suitable for the purchased kits (Qiagen, Hilden, Germany). Each reaction contained 0.5 µg of cDNA (for each mouse in the group; 3 or 4 mice per group).

Tumour tissue specimens were collected on day 21 after the inoculation of 4T1 cells from mice treated with calcitriol or its analogues. The control group received only the vehicle for vitamin D compounds. Calcitriol or its analogues downregulated the expression of 4 genes related to the Th1 response in tumour tissue (*Ifng* (only calcitriol), *Socs1*, *Tbx21* and *Fasl*) and upregulated 5 genes related to the Th2 response (*Ccl11*, *Ptgdr2*, *Il9*, *Asb2* and *Il5*). *Ccl7* and *Tmed*, associated with the Th2 response, were downregulated (Fig. 6, Supplemental Table 2). The expression of two genes associated with Th17 cells is intriguing since the *Il21* mRNA level decreased in tumour tissue from treated mice, whereas *Il17re* was increased. Seven genes related to Tregs were affected by treatment with calcitriol or its analogues; five of them were downregulated, i.e., *Fosl*, *Gata4, Hopx, Lrrc32,* and* Tnfrsf9*. Among the 2 upregulated genes, the most interesting was the increase in *Tnfsf11* (RANKL) expression by PRI-2191 (fold-change 3.798) and PRI-2205 (fold change 4.840) (Fig. 6, Supplemental Table 2). Moreover, the expression of *Perp* (TP53 apoptosis effector) was increased significantly in mice treated with calcitriol or its analogues, whereas *Stat1*, *Stat4* and *Stat6* were downregulated by calcitriol and PRI-2191.

**Figure 7.**
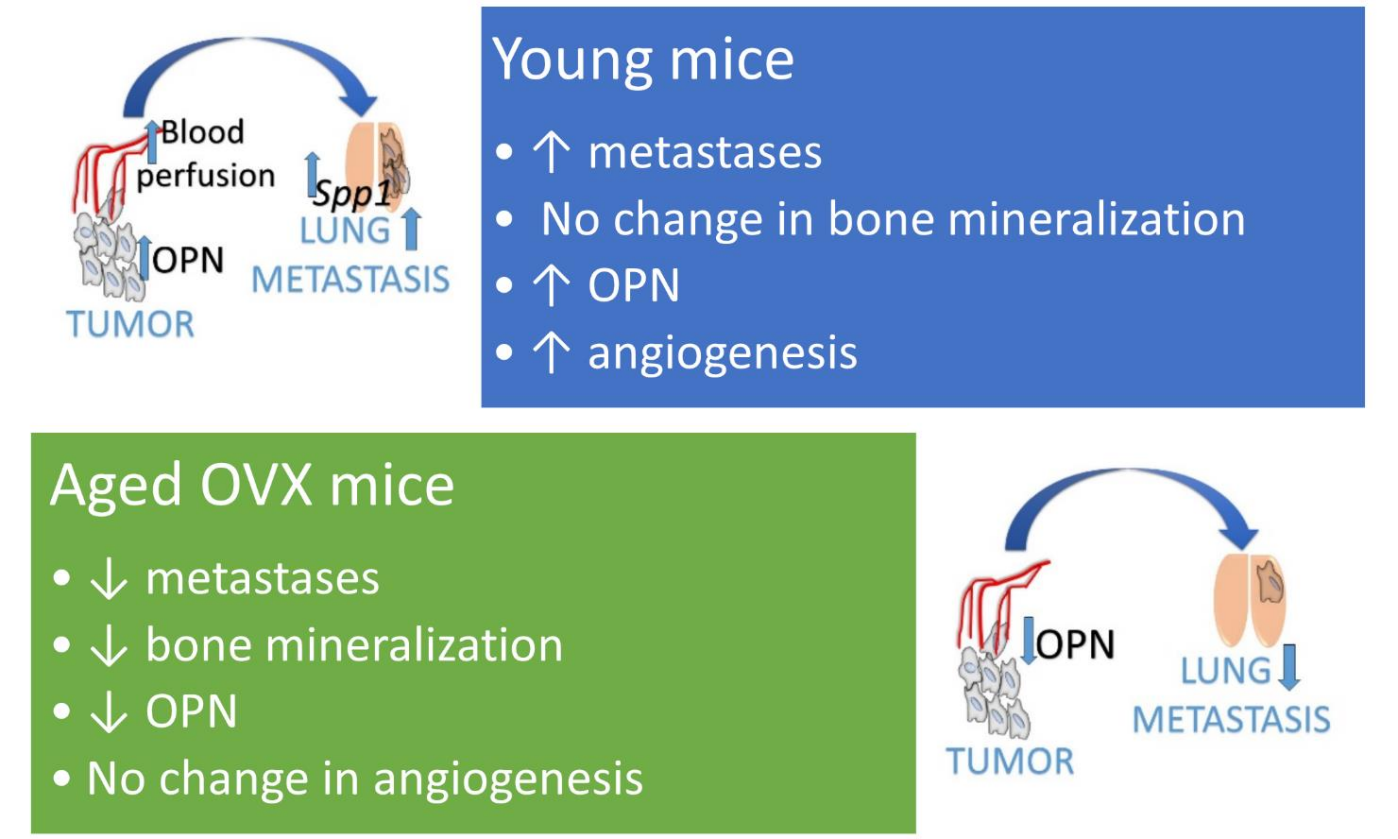
The main differences in the response to treatment with calcitriol and its analogues PRI-2191 and PRI-2205 between aged OVX and young mice bearing 4T1 breast cancer. Selected results of the analyses performed are presented. Young 6- to 8-week-old mice and aged 60-week-old ovariectomized (OVX) mice received an orthotopic injection of 4T1 mouse mammary gland cancer cells. Calcitriol (0.5 µg/kg), PRI-2191 (1 µg/kg) and PRI-2205 (10 µg/kg) were injected subcutaneously starting from day 7 and continuing three times a week to the end of the experiment.

## DISCUSSION

The calcitriol analogues used in our studies were previously selected as low toxic analogues with anticancer activity in colon, mammary gland and lung cancer models [17,24,43-45]. However, in our recent studies, we have shown that calcitriol and these two low calcaemic analogues, PRI-2191 (tacalcitol) and PRI-2205, stimulate the lung metastasis of 4T1 mammary gland tumours growing in young mice. This undesirable effect is due to the induction of a cancer permissive milieu [27,28]. In this study, we highlight that the same scheme of treatment used in aged OVX mice has different effects on lung metastasis and tumour angiogenesis than those previously shown in young mice (Fig. 7). In aged OVX mice, we have shown a transient decrease in lung metastasis on day 28 or a lack of pro-metastatic activity on day 33. This may result from the decrease in OPN expression and the lack of induction of angiogenesis in tumour tissue of these mice. Previously, in young mice, we have shown that tumour blood perfusion and tumour OPN level, as well as the *Spp1* mRNA level in lung tissue, were stimulated by treatment with calcitriol and its analogues, and there was increased metastatic potential of 4T1 cells [27,28]. OPN is a known cytokine with pleiotropic action towards accelerating tumour growth, angiogenesis and metastasis [46-48]. It also acts by increasing the immunosuppressive properties of regulatory T cells (Tregs) in the metastatic niche [49]. Therefore, the reduction in the levels of this cytokine by calcitriol and its analogues in tumour tissue and by analogues in the plasma may be responsible for the decreased lung metastatic potential of 4T1 cells in old OVX mice. Reduced OPN levels in tumour tissue after the administration of calcitriol or its analogues were rather unexpected because the OPN gene (*Spp1*) contains a vitamin D response element (VDRE) in the promoter region, whereby calcitriol stimulates various cells to secrete OPN [50-52]. On the other hand, T and B lymphocytes secrete OPN only after activation [53], and calcitriol is known to decrease the number of activated T and B cells [54,55]. The downregulation of *Stat1*, *Stat4* and *Stat6* observed in tumour tissue may confirm the reduced response to cytokines of lymphocytes and therefore may indicate a decrease in lymphocyte activation. STAT1 is necessary for IFNγ signalling, and STAT1 and STAT4 (IL-12) are the main factors for Th1 polarization. On the other hand, STAT6 stimulation by IL-4 is important for Th2 signalling [56-58]. Our gene expression analysis showed that many cytokines and their receptors were downregulated by calcitriol and its analogues in tumour tissue.

OPN also plays a crucial role in bone remodelling since it is involved in osteoclast chemotaxis and attachment to bone, as well as in osteoclast generation and osteoblast function [59]. According to this, decreased plasma and tumour tissue OPN levels may be connected to the observed deterioration of some bone mineralization parameters in aged mice. Vitamin D prevents the demineralization of bone. However, it has also been demonstrated that calcitriol induces bone resorption. This activity is realized through RANKL, whose expression is stimulated by calcitriol in osteoblasts, thus inducing osteoclastogenesis via the NF-κB pathway [60]. In our studies, we show that in tumour tissue, the expression of the RANKL gene (*Tnfsf11*) was increased by both calcitriol analogues but not by calcitriol. This upregulation correlated with increased osteoclastogenesis of bone marrow-derived macrophages from the same mice, which explains the impaired bone mineralization. On the other hand, calcitriol or some calcitriol analogues such as eldecalcitol (1α,25-dihydroxy-2β-(3-hydroxypropoxy) vitamin D_3_) or alfacalcidol (1α(OH)D_3_) are used in the treatment of osteoporosis because they increase bone mineralization by reducing bone resorption [61,62] through the suppression of RANKL expression in osteoblasts and decreased osteoclastogenic potential, as shown in eldecalcitol- or alfacalcidol-treated mice [63,64]. Moreover, Starczak et al. evaluated a conditional deletion mouse model, Ctsk^Cre^/Vdr^-/-^, and showed that the absence of VDR in mature osteoclasts causes enhanced bone loss in young and OVX mice through increased osteoclastic activity without increased osteoclastogenesis [65]. Therefore, it has been shown that calcitriol itself and analogues such as eldecalcitol exert anti-osteoporotic activity through VDR.

Our further studies, however, produced contradictory results. Whereas *Tnfsf11* expression in tumour tissue was increased by the two calcitriol analogues, RANKL was not affected at the protein level in tumour tissue but was increased by PRI-2191 in the plasma (p=0.0889). PRI-2191 also seemed to increase plasma osteocalcin levels (*p*=0.0933). This might suggest different posttranscriptional mechanisms in tumours compared to other tissues. Moreover, increased RANKL and osteocalcin and decreased OPN plasma concentrations suggest that calcitriol analogues induce intensive, ongoing bone remodelling in old OVX mice bearing 4T1 breast cancer.

PRI-2191 and PRI-2205 [66] have a higher binding affinity for vitamin D binding protein (DBP) than does calcitriol. However, PRI-2205 has a higher binding affinity for VDR than do calcitriol and PRI-2191 [66], whereas the affinity of PRI-2191 for VDR is similar to that of calcitriol [67]. We have previously shown that PRI-2205 possesses a higher binding affinity not only for VDR and DBP but also for the CAR/RXR (constitutive androstane receptor/retinoid X receptor) ligand binding domain. This may, in part, explain its very low toxicity and sustained anticancer activity [68], as well as the different mechanisms of action in comparison to PRI-2191 and calcitriol [39,66,68]. It may also explain why PRI-2205 did not affect vitamin D metabolite levels, as shown in our recent studies, whereas calcitriol and PRI-2191 decreased the levels of 25(OH)D_3_ and 24,25(OH)_2_D_3_ in aged OVX mice. Calcitriol and PRI-2191 are metabolized to inactive metabolites through CYP24 [69], a target of VDR [70]. The transactivation of responsive elements in CYP24 by the VDR/RXR complex leads to increased expression of CYP24 and reduced levels of the biologically active calcitriol [70]. Moreau *et al.* showed that CAR binds and transactivates VDREs present in the CYP24 promoter. They also established that a specific human CAR agonist, CITCO, increases CYP24 mRNA expression in primary human hepatocytes. These findings suggest that VDR-CAR crosstalk resulting from the recognition of the same response elements is reciprocal [70]. Such crosstalk provides, at least in part, an explanation for the observed differences in the levels of vitamin D metabolites, which are similar in calcitriol- and PRI-2191-treated mice but different in PRI-2205-treated mice. However, studies have shown that calcipotriol (with the same side chain modification as PRI-2205) may share catabolic enzymes with calcitriol [71]. On the other hand, the affinity of calcipotriol for VDR is similar to that of calcitriol, but it is 30-fold lower for DBP [72]. Therefore, we can speculate that the highest DBP, VDR and CAR/RXR binding among all tested compounds may alter the metabolic pathway of PRI-2205.

Up to the present, young mice inoculated with various tumour cells were commonly used as an *in vivo* model for structure-activity studies of vitamin D_3_ analogues as potential anticancer agents. It is worth emphasizing that when we measured plasma levels of 25(OH)D in young 4T1 tumour bearing mice, both calcitriol and PRI-2191 decreased their levels (similar to what was observed in the current study), but PRI-2205 revealed similar activity [27] that was not observed in OVX mice. These results indicate that the profile of activities of calcitriol and its analogues differ based on age. In Figure 7, we summarize the differences detected during our studies between young [27,28] and aged OVX mice bearing 4T1 tumours. In conclusion, in-depth metabolic pathway studies, as well as studies on anticancer activity and analyses of bone metabolism in old mouse models, are needed before considering new vitamin D analogues with various chemical modifications as effective drugs supporting anticancer treatment, especially in the elderly.

## Supplementary Materials

The Supplemenantry data can be found online at: www.aginganddisease.org/EN/10.14336/AD.2018.0921
