# Supplementary material for: Calcitriol Analogues Decrease Lung Metastasis but Impair Bone Metabolism in Aged Ovariectomized Mice Bearing 4T1 Mammary Gland Tumours
Source: Aging Dis. 2019 Oct 1;10(5):977–91. doi: 10.14336/AD.2018.0921 (PMC6764735; doi:10.14336/AD.2018.0921)
Supplement: Supplementary file 1 — The Supplemenantry data can be found online at: www.aginganddisease.org/EN/10.14336/AD.2018.0921 [file AD-10-5-977-s.pdf]

SUPPLEMENTARY DATA

Calcitriol Analogues Decrease Lung Metastasis but Impair Bone Metabolism in Aged Ovariectomized Mice Bearing 4T1 Mammary Gland Tumours

Artur Anisiewicz[1,#], Beata Filip-Psurska[1,#], Agata Pawlik[1,#], Anna Nasulewicz-Goldeman[1], Tomasz Piasecki[2], Konrad Kowalski[3], Magdalena Maciejewska[1], Joanna Jarosz[1], Joanna Banach[1], Diana Papiernik[1], Andrzej Mazur[4], Andrzej Kutner[5], Jeanette A. Maier[6], Joanna Wietrzyk[1,*]

[1]Department of Experimental Oncology, Hirszfeld Institute of Immunology and Experimental Therapy, Wroclaw, Poland.
[2]Faculty of Veterinary Medicine, Wroclaw University of Environmental and Life Sciences, Wroclaw, Poland.
[3]Masdiag Sp. z o.o., Żeromskiego 33, Warsaw, Poland.
[4]Université Clermont Auvergne, INRA, UNH, Unité de Nutrition Humaine, F-63000 Clermont-Ferrand, France.
[5]Pharmaceutical Research Institute, 01-793 Warsaw, Poland.
[6]Università di Milano, Dept. Biomedical and Clinical Sciences, 20157 Milano, Italy

© 2017. Anisiewicz A et al. Published online at http://www.aginganddisease.org/EN/10.14336/AD.2018.0921

SUPPLEMENTARY DATA

Supplemental Table 1. List of genes included in *Mouse T Helper Cell Differentiation RT^2 Profiler Array*.

Symbol	Description
Asb2	Ankyrin repeat and SOCS box-containing 2
Cacna1f	Calcium channel, voltage-dependent, alpha 1F subunit
Ccl11	Chemokine (C-C motif) ligand 11
Ccl5	Chemokine (C-C motif) ligand 5
Ccl7	Chemokine (C-C motif) ligand 7
Ccr3	Chemokine (C-C motif) receptor 3
Ccr4	Chemokine (C-C motif) receptor 4
Ccr6	Chemokine (C-C motif) receptor 6
Cebpb	CCAAT/enhancer binding protein (C/EBP), beta
Chd7	Chromodomain helicase DNA binding protein 7
Csf2	Colony stimulating factor 2 (granulocyte-macrophage)
Fasl	Fas ligand (TNF superfamily, member 6)
Fosl1	Fos-like antigen 1
Foxp3	Forkhead box P3
Gata3	GATA binding protein 3
Gata4	GATA binding protein 4
Gfi1	Growth factor independent 1
Ptgdr2	G protein-coupled receptor 44
Havcr2	Hepatitis A virus cellular receptor 2
Hopx	HOP homeobox
Hoxa10	Homeobox A10
Hoxa3	Homeobox A3
Icos	Inducible T-cell co-stimulator
Id2	Inhibitor of DNA binding 2
Ifng	Interferon gamma
Igsf6	Immunoglobulin superfamily, member 6
Ikzf2	IKAROS family zinc finger 2
Il12b	Interleukin 12B
Il12rb2	Interleukin 12 receptor, beta 2
Il13	Interleukin 13
Il13ra1	Interleukin 13 receptor, alpha 1
Il17a	Interleukin 17A
Il17re	Interleukin 17 receptor E
Il18	Interleukin 18
Il18r1	Interleukin 18 receptor 1
Il18rap	Interleukin 18 receptor accessory protein
Il1r1	Interleukin 1 receptor, type I
Il1r2	Interleukin 1 receptor, type II
Il1rl1	Interleukin 1 receptor-like 1
Il2	Interleukin 2
Il21	Interleukin 21
Il2ra	Interleukin 2 receptor, alpha chain
Il4	Interleukin 4
Il4ra	Interleukin 4 receptor, alpha
Il5	Interleukin 5
Il9	Interleukin 9
Irf1	Interferon regulatory factor 1
Irf4	Interferon regulatory factor 4
Irf8	Interferon regulatory factor 8
Jak1	Janus kinase 1
Lrrc32	Leucine rich repeat containing 32
Maf	Avian musculoaponeurotic fibrosarcoma (v-maf) AS42 oncogene homolog
Myb	Myeloblastosis oncogene
Nfatc1	Nuclear factor of activated T-cells, cytoplasmic, calcineurin-dependent 1
Nfatc2	Nuclear factor of activated T-cells, cytoplasmic, calcineurin-dependent 2
Nfatc2ip	Nuclear factor of activated T-cells, cytoplasmic, calcineurin-dependent 2 interacting protein

© 2017. Anisiewicz A et al. Published online at http://www.aginganddisease.org/EN/10.14336/AD.2018.0921

SUPPLEMENTARY DATA

Nr4a1	Nuclear receptor subfamily 4, group A, member 1
Nr4a3	Nuclear receptor subfamily 4, group A, member 3
Perp	PERP, TP53 apoptosis effector
Pkd2	Polycystic kidney disease 2
Pou2f2	POU domain, class 2, transcription factor 2
Pparg	Peroxisome proliferator activated receptor gamma
Rel	Reticuloendotheliosis oncogene
Rora	RAR-related orphan receptor alpha
Rorc	RAR-related orphan receptor gamma
Runx1	Runt related transcription factor 1
Runx3	Runt related transcription factor 3
Socs1	Suppressor of cytokine signaling 1
Socs5	Suppressor of cytokine signaling 5
Stat1	Signal transducer and activator of transcription 1
Stat4	Signal transducer and activator of transcription 4
Stat6	Signal transducer and activator of transcription 6
Tbx21	T-box 21
Tgif1	TGFB-induced factor homeobox 1
Tlr4	Toll-like receptor 4
Tlr6	Toll-like receptor 6
Tmed1	Transmembrane emp24 domain containing 1
Tnf	Tumor necrosis factor
Tnfrsf9	Tumor necrosis factor receptor superfamily, member 9
Tnfsf11	Tumor necrosis factor (ligand) superfamily, member 11
Trp53inp1	Transformation related protein 53 inducible nuclear protein 1
Uts2	Urotensin 2
Zbtb7b	Zinc finger and BTB domain containing 7B
Zeb1	Zinc finger E-box binding homeobox 1

Supplemental Table 2. Fold change values of genes associated with mouse T helper cell differentiation in tumour samples from 4T1 tumour bearing mice treated with calcitriol or its analogues PRI-2191 and PRI-2205.

Tumour tissue						
**Gene**	**Calcitriol**		**PRI-2191**		**PRI-2205**	
	**Mean**	**SD**	**Mean**	**SD**	**Mean**	**SD**
Asb2	2.674	1.600	1.224	0.737	1.410	0.792
Cacna1f	1.264	0.542	1.612	0.514	1.099	0.318
Ccl11	2.094	1.646	2.061	1.826	3.438	2.277
Ccl5	0.694	0.226	0.913	0.508	1.305	0.538
Ccl7	0.735	0.140	0.609	0.111	0.808	0.138
Ccr3	0.932	0.198	0.756	0.203	0.879	0.209
Ccr4	1.128	0.274	1.118	0.213	1.398	0.335
Ccr6	5.510	5.185	7.018	10.445	3.269	2.945
Cebpb	0.838	0.068	0.978	0.075	0.936	0.077
Chd7	0.780	0.061	0.942	0.077	1.375	0.118
Csf2	1.985	0.418	1.401	0.399	1.306	0.296
Fasl	0.694	0.232	0.504	0.215	1.216	0.575
Fosl1	0.666	0.268	0.676	0.171	1.064	0.280
Foxp3	1.266	0.573	1.417	0.463	1.274	0.470
Gata3	0.776	0.052	0.728	0.052	1.149	0.071
Gata4	0.630	0.110	1.028	0.193	1.117	0.198
Gfi1	1.105	0.605	1.665	0.816	1.476	0.489
Ptgdr2	1.589	0.775	2.296	0.977	3.400	1.885

© 2017. Anisiewicz A et al. Published online at http://www.aginganddisease.org/EN/10.14336/AD.2018.0921

Havcr2	0.879	0.253	1.133	0.339	0.910	0.236
Hopx	0.640	0.175	0.822	0.290	0.938	0.297
Hoxa10	1.379	0.161	1.095	0.116	1.529	0.179
Hoxa3	0.725	0.052	0.623	0.062	1.119	0.072
Icos	0.795	0.387	0.961	0.457	1.320	0.735
Id2	0.983	0.408	1.021	0.508	0.779	0.325
Ifng	0.594	0.273	1.178	0.762	1.982	1.496
Igsf6	0.837	0.283	0.787	0.255	0.790	0.269
Ikzf2	1.058	0.150	0.606	0.131	1.114	0.202
Il12b	1.153	0.581	0.839	0.492	2.059	1.274
Il12rb2	1.173	0.501	1.301	0.482	2.708	1.004
Il13	1.510	0.440	1.933	0.464	1.397	0.333
Il13ra1	1.865	0.505	0.920	0.129	0.898	0.117
Il17a	0.815	0.135	1.271	0.225	1.911	0.401
Il17re	5.205	1.105	1.011	0.248	4.802	1.318
Il18	1.057	0.146	0.944	0.124	1.098	0.143
Il18r1	1.061	0.428	1.201	0.541	1.122	0.298
Il18rap	1.580	1.148	1.109	0.635	1.865	1.965
Il1r1	0.810	0.120	0.709	0.097	0.909	0.140
Il1r2	1.352	1.108	1.197	0.596	0.892	0.485
Il1rl1	1.390	0.714	1.251	0.490	2.234	0.973
Il2	0.980	0.254	1.578	0.375	1.905	0.558
Il21	0.671	0.254	0.817	0.186	1.216	0.434
Il2ra	0.827	0.244	1.046	0.309	0.938	0.231
Il4	0.969	0.250	1.015	0.383	0.904	0.276
Il4ra	0.797	0.110	0.808	0.117	1.061	0.125
Il5	1.616	1.307	2.619	1.890	1.824	1.366
Il9	3.534	2.300	2.045	1.567	4.154	3.750
Irf1	0.709	0.046	0.755	0.053	0.831	0.048
Irf4	1.230	0.451	1.942	0.946	1.680	0.855
Irf8	0.778	0.108	1.114	0.162	1.026	0.138
Jak1	0.867	0.060	1.097	0.071	0.998	0.065
Lrrc32	0.680	0.191	1.126	0.395	0.987	0.237
Maf	0.879	0.204	0.913	0.176	1.050	0.184
Myb	1.689	0.619	1.394	0.428	2.895	1.287
Nfatc1	0.775	0.071	0.791	0.084	0.861	0.072
Nfatc2	1.077	0.189	0.961	0.159	1.080	0.184
Nfatc2ip	0.795	0.096	0.870	0.095	1.147	0.126
Nr4a1	0.745	0.087	0.784	0.090	0.887	0.086
Nr4a3	0.701	0.263	0.780	0.279	1.144	0.477
Perp	2.566	0.682	1.826	0.631	3.476	2.073
Pkd2	0.734	0.027	0.628	0.026	0.898	0.032
Pou2f2	1.215	0.357	1.355	0.453	1.706	0.536
Pparg	1.829	0.656	4.033	1.631	1.526	0.456
Rel	0.971	0.214	0.881	0.183	1.124	0.216
Rora	1.080	0.230	1.329	0.288	1.468	0.312
Rorc	1.137	0.103	1.300	0.106	1.215	0.102
Runx1	0.786	0.052	0.693	0.046	0.930	0.055
Runx3	0.733	0.141	0.694	0.142	1.162	0.230
Socs1	0.689	0.128	0.932	0.256	0.967	0.194
Socs5	1.184	0.231	1.169	0.223	1.253	0.210
Stat1	0.583	0.096	0.731	0.189	1.121	0.324
Stat4	0.653	0.191	0.836	0.410	1.338	0.590
Stat6	0.690	0.115	1.027	0.234	1.581	0.236
Tbx21	0.589	0.183	1.213	0.466	1.296	0.404
Tgif1	0.989	0.253	0.595	0.171	1.090	0.322
Tlr4	0.949	0.214	1.072	0.220	1.019	0.209
Tlr6	0.984	0.473	0.677	0.299	0.907	0.409
Tmed1	0.679	0.062	0.599	0.058	0.806	0.064

© 2017. Anisiewicz A et al. Published online at http://www.aginganddisease.org/EN/10.14336/AD.2018.0921

Tnf	0.834	0.182	0.518	0.133	1.609	0.752
Tnfrsf9	0.476	0.123	0.572	0.090	0.550	0.094
Tnfsf11	1.529	0.528	3.798	1.707	4.840	3.454
Trp53inp1	1.072	0.226	0.725	0.131	0.902	0.182
Uts2	0.716	0.328	0.692	0.250	1.135	0.480
Zbtb7b	0.840	0.078	0.854	0.085	1.024	0.088
Zeb1	0.849	0.155	1.325	0.257	1.204	0.283

Tumour tissue specimens were collected on the day 21 (after inoculation with 4T1 cells) from 60 week old mice treated with calcitriol or its analogues and control group receiving vehicle for vitamin D compounds. *Mouse T Helper Cell Differentiation RT^2 Profiler Array* (Qiagen, Hilden, Germany) was used for Real-time PCR screening (84 key genes and 5 housekeeping genes). Data shows a mean fold change values and standard deviation for each group. Fold-change (RQ) of target genes was defined using double delta Ct method in reference to the heat shock protein 90 alpha (cytosolic), class B member 1 (*Hsp90ab1*) for tumour samples. Then the results were adjusted to the values obtained for the control group within the day 21 of the experiment for each treatment group. Data analysis was acquired using Qiagen online software (Qiagen, Hilden, Germany). PCR amplification cycles were as follows 95°C for 10 s and 58°C for 45 s (50 cycles). Each reaction contained 0.5 µg of cDNA (for each mouse in the group; 3 or 4 mice per group).

© 2017. Anisiewicz A et al. Published online at http://www.aginganddisease.org/EN/10.14336/AD.2018.0921
